# The Bandage as Used by Professor Dudley and His Proselytes

**Published:** 1880-03

**Authors:** F. L. Merriwether

**Affiliations:** Houston, Texas


Medical and Surgical Journal.

Vol. XVII.] MARCH—1880. [No. 12.

### Original Communications.

### THE BANDAGE AS USED BY PROFESSOR DUDLEY AND HIS PROSELYTES.

By F. L. MERRIWETHER, Houston, Texas.

Mr. Editor—I wish to give to the public, my experience in the use of the bandage as taught by Professor B. W. Dudley, of Lexington, Kentucky, years ago. Old remedies are sometimes abandoned by the profession for want of proper skill in their application, which, I think, is one reason why the bandage has been so little used notwithstanding its acknowledged potency in the treatment of many surgical cases. I will here make a few remarks extracted from notes taken from Dudley's lectures, by J. M. Bush, adjunct professor to Dudley, which was published in the *Transylvania Medical Journal*, volume X, number 1, viz.: "The fact is most extensively known to the profession at large, and particularly familiar to the practicioner of the great West, over whose immense territory his private pupils are scattered, that Professor Dudley's name and the bandage are intimately associated, and may I not even say in earnest reality, almost to the exclusion of any other name? There must be a reason for this distinction, and a very good cause for his warm and zealous support of a remedy, so much a favorite in his skillful hands.

An experience of thirty years, must be sufficient to afford ample time to prove the successful triumph of its applications to various forms of disease, and give a thorough knowledge of its influence in controlling different troubles of the body, as well as a familiar acquaintance with its relations to the laws of vitality. The charm-like potency with which it controls inflammation through its various forms of adhesive ulcerative, abortive, secretive or granulative action, we have often witnessed in very many interesting cases.

For sanative purposes, it is a means most excellent and certain, in exciting necessary inflammation or for subduing the same action, when the surgeon with that view makes its application. It is useful for the discussion of swellings, by promoting absorption; for the production or restoration of secretion; for the direct alleviation of acute pain, by its surprising effects upon the nerves; for bidding defiance to the laws of life itself by subduing, in the muscular system, its power of contractility, thus becoming the most efficient remedy in almost all fractures, obviating the necessity, most completely, for all apparatus presented to the surgeon in such extensive profusion of boxes, splints, inclined planes, etc., and all other fixtures so admirably calculated, not only for the inconvenience of the practitioner, but for the annoyance of the patient; for its magic power in re-producing lost parts, thereby preventing a loss of the symmetry of the body; for preparing parts about to be submitted to capital operations, by placing them in a proper condition to experience less pain; and for the more expert employment of instruments, at the same time its influence causing more speedy restoration of the proper condition. Limbs that have been condemned by surgeons as incurable, and consigned to the amputating knife, have been rescued from the operation, and now exist healthy and restored, living monuments of the unrivalled success and unprecedented superiority of graduated compression.

The prominent object of these very limited remarks, is to detail a number of dissimilar cases treated by Professor Dudley. First, with a view of illustrating its happy effects in inflammation as that process is manifested in its progress and termination, in adhesion, ulceration, granulation, suppuration, resolution, cicatrization and mortification.

Case 1. A gentlemen, in consequence of a fracture of his leg and the bad surgery in his treatment, suffered from the influence of numerous abscesses, embeded in and extending through the superficial and deep muscles of the entire extremity. His case was managed for a long time by the application of all the usual means recommended by the acknowledged surgery of the day, yet no alleviation of the malady would present itself. The suppurative cavities were continually discharging the most copious and exhausting secretions and it was determined by his attendant that amputation of the limb was the only cure, and even in this conclusion they failed to secure their own confidence in the result, thinking that in the removal of the limb, some portions of the burrowed ulcers would involve the stump, the ulcers extending up to the groin. The patient was consequently sent to Dudley to amputate the limb. But the Professor had too much confidence in the compressing roller to make this sacrifice to the knife and saw. A bandage was applied from the end of his toes to the knee, securing compresses over the sinews of the leg. Other graduated compresses of sufficient magnitude were now adjusted directly over the abscesses, and another broader roller being secured around the hips, was brought down over the thigh to the knee where the first bandage terminated at the points where the puss was discharging; causing the compresses to obliterate the cavities and thus secure immediate apposition of their walls, thus completing the dressings. This patient was dressed twice again in the same manner and at the end of three weeks his cure was complete.

Case 2. A young man, æt twenty-two years, was engaged dressing mill stones in an old powder establishment, a large quantity of powder was scattered both about and under the floor. A spark flew from his peck and ignited the powder and caused an explosion. He received an extensive burn; the head face and hands, as far as half way up the forearm, sustaining the greatest injury. The hands and wrist were deeply burned. All the skin was drawn off, with the nail, as perfectly and completely as if it were a glove, from both hands. The different applications of linseed oil, cotton, and delicate cerates, with other usual agents, were resorted to. In a few days both hands and wrists were most luxuriently granutating, and suppurating copiously. It required an hour to dress them, while the sufferer endured most agonizing pain, the intensity of the morbid sensibility seeming daily to threaten violent and general convulsion. The bandage was advised, a piece of thin calico was selected, out of which rollers for the thumb and fingers were constructed. These were applied firmly from the end to the hand; then another bandage of greater width was adapted, commencing at the digital extremities, embracing all of the fingers, and carried up the hands and wrist to near the elbow.

A sponge and warm water was frequently applied to remove the pus, as it exuded through the dressing, which was not disturbed until from absorption, they had become very loose; half dozen applications of the same kind and the patient was cured. In this interesting case the compression merits the highest admiration.

While it obviated frequent and painful dressings, the tendency of which would have been to prolong the treatment, it sustained the granulation and suppuration, which had so abundantly occurred. The instantaneous effect was a cessation of pain, and had immediate control over the granulation and suppurative inflammation. All the integuments being destroyed, there was nothing to prevent the spouting of recently formed flesh, as it was presented in the profuse granulations, and nature must have become exhausted from the pain and discharge together before she could have been prepared for cicatrization of the extensive surfaces. The bandage was substituted for the most natural protection of the parts—the skin, and while it then acted in excluding the painful influence of the atmosphere, it at the same time controlled the nerves by pressure; the granulations were all leveled, and their secreting surfaces rapidly induced to withhold suppuration and to protect themselves by cicatrization, all of which was accomplished in an incredibly short time.

Case 3. A female of intemperate habits, during a drunken fit, sustained a severe comminuted fracture of the ankle, with extensive injury of the soft parts, resulting in the death of the foot. Professor Dudley was called to the case as the operating surgeon. Finding the leg œdematous, and the sphacelus progressing along the continuity of the leg, he declined using the knife until a more suitable condition of the parts could be procured. To effect this object the bandage was applied from the toe to the knee, thus embracing both the dead and the living parts. As a simple and cleanly dressing nothing could have been better for the mortified foot and ankle, while its power over the absorbents of the limb was believed and understood, the dropsy rapidly disappearing through the lymphatics, thus making the parts more healthy for the knife. At the same time the line of demarkation was distinctly defined, isolating the living from the dead parts. In this instance the mortification was caused to move hastily and define itself by pressure. Professor Dudley does not adopt the principals of John Hunter—to wait on nature for the line of separation in mortification—but with Larrey operates while it is progressing, other circumstances not forbidding.

Case 4. In an affair of honor a gentlemen received his antagonist's ball in the right thigh, opening the fermoral artery, then passed through the left thigh, wounding the sciatic nerve. In an instant the surgeon (Professor Dudley) placed his thumb over the divided artery where it passed over the pubis, completely arresting the hemorrhage, while it rescued for a time the patient's life. An assistant changed thumbs with the professor. A bandage was commenced at the toe, and continued within two or three inches of the wound; large compresses were then placed over the artery and along its course, extending above and below the points of division some two or more inches, when the roller was applied, binding firmly the pledgets until it reached the hips around which it was fixed. The man was then removed home, which was some distance, where it was designed to tie the fermoral artery. It was night, and no bleeding having occurred, not even enough to moisten the bandage, it was determined to wait until morning to operate. Morning arrived, and no manifestations appeared that the dressings were insecure; the dressings were undisturbed, and so they continued for eleven days, when the bandages on the first was cut away with a view to examine the interior tibial artery as it passed over the instep. No pulsation in the vessel could be detected, yet pressure with the finger left a white spot that was soon reddened again by the circulation. On the 13th day the posterior tibial artery was examined, and gave distinct evidence of circulation. All the dressings were now removed, and the entire wound found to be united and healed with the exception of the side where the ball passed out. This was granulating and discharged pus.

We record this case to show that pressure is completely competent, with the aid of the compress, even in injuries of large arteries, to supersede the necessity of the ligature. It brought into contact the severed tunic of the blood vessel, and so maintained the, at the same time excited the process of adhesive inflammation by which the artery was completely sealed, and the cure perfected. Small arteries on the arm or leg are easily secured under the compress and bandage, without resorting to the knife or ligature.

Case 5. A gentlemen was thrown from his horse and sustained an extensive compound fracture of his ankle. In the lacerated injury the anterior tibial artery was wounded and presented in a short time an aneurism of considerable magnitude. A thick pledget of cotton cloth was placed on the pulsating tumor, extending some inch or two above and below it; the bandage was then applied, commencing at the toes, extending up to the knee and embracing firmly the pledgets, the remedy thus being applied two or three times in twenty days the cure was complete. Here, while the compress, secured by the roller, arrested the circulation through the artery, effecting a stagnation and coagulation of blood in the sac it, by its mechanical influence, prevented all swelling of the foot, ankle and leg. Nor does it appear at all paradoxical to assert that it excited the absorbants to destructive action in the discussion of the tumor, by the removal of coagulated blood and extraneous parts.

The above cases will suffice, for the present, to show the power of the bandage, as a surgical agent, in arresting diseased action. I will now relate a few cases of my own, to show its potency to heal ulcers, etc.

A gentleman of this county by the name of John Vincent, had an ulcer of long standing, (his age was about forty years.) He came to me to have it cured; there was so much surface denuded by the ulcerative process that Ithought proper to measure its length and breadth. It was situated on his right leg about half way between the ankle and knee, on the inside of the leg, and was 5 inches long and $4\frac{1}{2}$ wide; his constitution was anemic; had had syphilis. He told me that a good many physicians had tried to cure his ulcer but had failed. I put him upon constitutional treatment, alterative tonics, with the muriated tincture of iron, and applied the bandage from his toes to his knee as tight as he could bear it, after first applying a solution of carbolic acid to destroy the intolerable bad smell and excite healthy action; then put ginned cotton as lint, spread very thin, over the sore and a plaster of common salve made of equal parts of beeswax and tallow and one-third soft turpentine from the tree, spread upon a piece of newspaper for convenience and economy. In a few day's the ulcer began to put on a healthy aspect, shoot out granulations and was relieved of all bad smell. I alternated the carbolic with diluted nitric acid, made strong enough to excite some smarting pain when applied. The ulcer continued to heal until it was finally skinned over. I think it took about two months to complete the cure. The dressing was applied daily. I have had a great many cases and whenever the ulcer is between the joints, will always insure the cure. I dress them but once daily.

I have been using the bandage for fifty years and can safely say it never has disappointed me. I use it in all cases of fractures with success, but it does require some considerable tact and experience to adjust it properly. Calico is the material I always used to make the bandages; two, three and four inches, is the proper width.

In dressing the arm, the turns of the roller should uniformly be from the ulner to the radial side, in front. On the foot and leg the turns should be from the inner to the outer side of the limb. Great pains should be taken to adapt it equally smooth and firmly, not with a horse power, but, I would say as a general remark, with force of two or three pounds. Care must certainly be taken not to draw too tightly around the ankle or the wrist, because here the parts are nearly all tendons and do not require it; and moreover, undue pressure is very apt to excite intolerable pain in consequence of its ligature effect. Under these circumstances, over these parts, it is enough to carry it with smoothness and gentle firmness, increasing the force as it ascends the muscular portion of the limb.

The good effects of the remedy are greatly facilitated in most of the cases to which it is applied, especially those involving inflammation, by keeping the bandage continually bathed in warm water or spirits. Constitution, age and sex will always, in every case, influence the intelligent physician as to the force with which he will make the compression. A delicate and excitable female would not bear the same degree as a robust, phlegmatic man, while that which would be suitable with either, would certainly cause destructive ulceration with the infant. The bandage should always be divested of the selvidge, and then wet and rolled up tight, and put on while wet. It then adapts itself properly to all parts of the limb. I make these remarks for the benefit of the young surgeons, as scarcely anything relative to Professor Dudley's notoriety about the use of the bandage has ever been published that has come to my knowledge. I received my first surgical knowedge from attending his lectures in the years 1877 and '78, and have been in active practice ever since, and herewith endorse all he says upon the efficacy of the bandage.

